# Supplementary material for: Continuity of care interventions for people with stroke and their caregivers: A scoping review protocol
Source: PLoS One. 2025 May 9;20(5):e0323344. doi: 10.1371/journal.pone.0323344 (PMC12063880; doi:10.1371/journal.pone.0323344)
Supplement: S1 Appendix — (DOCX) [file pone.0323344.s001.docx]

**S1 Appendix A**

**Search strategy for MEDLINE via Ovid**

1. (stroke or cerebrovasc$ or brain vasc$ or cerebral vasc$ or cva$).tw,kf.
2. ((brain$ or cerebr$ or cerebell$ or vertebrobasilar or hemispher$ orintracran$ orintracerebral orinfratentorial or supratentorial or MCA or anterior circulation or posterior circulation or basal ganglia) adj1 (isch?emi$ or infarct$ or thrombo$ or emboli$)).tw,kf.
3. ((brain$ or cerebr$ or cerebell$ or intracerebral or intracran$ or parenchymal or intraventricular or infratentorial or supratentorial or basal gangli$) adj1 (haemorrhage$ or hemorrhage$ or haematoma$ or hematoma$ or bleed$)).tw,kf.
4. (early supported discharge or ESD).tw,kf.
5. ((early or earlier or prompt or accelerate$ or acute or subacute or supported) adj3 discharg$).tw,kf.
6. (return$ adj2 home$).tw,kf.
7. (hospital$ adj2 home$).tw,kf.
8. (rehab$ adj2 home$).tw,kf.
9. (acute$ adj2 home$).tw,kf.
10. (post-discharge adj4 (support$ or care)).tw,kf.
11. (patient adj2 (discharge or transition$ or navigat$)).tw,kf.
12. (follow-up care or follow-up after discharge or care-coordinati$ or discharge plan$).tw,kf.
13. (care adj2 (contin$ or transition$ or navigat$)).tw,kf.
14. (stroke adj3 (discharge or transition$ or navigat$)).tw,kf.
15. 1 or 2 or 3
16. 4 or 5 or 6 or 7 or 8 or 9 or 10 or 11 or 12 or 13 or 14
17. 15 and 16
18. limit 17 to (english language and yr="2000 -Current")
