# Supplementary material for: Continuity of care interventions for people with stroke and their caregivers: A scoping review protocol
Source: PLoS One. 2025 May 9;20(5):e0323344. doi: 10.1371/journal.pone.0323344 (PMC12063880; doi:10.1371/journal.pone.0323344)
Supplement: S2 Appendix — (DOCX) [file pone.0323344.s002.docx]

**S2 Appendix B**

**Draft Interview Guide**

Checklist for interviewer:

Before introducing the questions, ensure that the following has taken place:

1. Explain the purpose of the interview (below).
2. Review the informed consent form, emphasizing that participation is voluntary, and information will be kept confidential. For interviews done over the phone or zoom, the interview will be recorded and stored on a secure, encrypted network.
3. Use prompts like “can you give me an example?” or “please explain” to gain more insight into the topics.

Interview

The purpose of this interview is to hear your feedback about the findings from our scoping review. We will begin with first showing some slides that outline the current results of the scoping review. Following the presentation of results, we will ask you a series of questions:

1. What are your thoughts on the findings?
2. How do these findings help you or others?
3. Where do you get new information relating to continuity of care?
4. What do you like best about the proposed scoping review results?
5. Have we missed anything?
6. How useful would this be to future continuity of care interventions?
7. What recommendations do you have for improving this scoping review?

Interviewer:

Please thank the participant for sharing their experiences and thoughts with you today. Ask if they have any further questions or comments they would like to share. Ask if you could contact them in the future if you have any clarifying questions.

The questions seen above are developed from the following sources^1^

1. Krueger RA, Casey MA. Designing and conducting focus group interviews: Citeseer 2002.
