# Supplementary material for: Continuity of care interventions for people with stroke and their caregivers: A scoping review protocol
Source: PLoS One. 2025 May 9;20(5):e0323344. doi: 10.1371/journal.pone.0323344 (PMC12063880; doi:10.1371/journal.pone.0323344)
Supplement: Attachment — Submitted filename: Reviewed PONE-D-24-30063_reviewer.pdf [file pone.0323344.s004.pdf]

PLOS ONE

Continuity of Care Interventions for People with Stroke and their Caregivers: A Scoping Review Protocol

--Manuscript Draft--

**Manuscript Number:**	PONE-D-24-30063
**Article Type:**	Study Protocol
**Full Title:**	Continuity of Care Interventions for People with Stroke and their Caregivers: A Scoping Review Protocol
**Short Title:**	Continuity of Care Interventions for People with Stroke and their Caregivers
**Corresponding Author:**	Jill I Cameron<br>University of Toronto<br>Toronto, CANADA
**Keywords:**	continuity of care; stroke; people with stroke; caregiver; Scoping review; family-centered care
**Abstract:**	Background: The needs of people with stroke and caregivers' change as they transition across the stroke care continuum from initial symptom onset to community living. Continuity of care interventions may support these changing needs. Continuity of care refers to care provided to individuals that is coherent, connected, and consistent across settings. Interventions have been developed to enhance continuity of care, but to date, no reviews have identified the core components of these interventions specific to various care transitions.<br>Objective: To examine the literature on continuity of care interventions that address the needs of PWS and/or their caregivers specific to stroke care transitions.<br>Methods: The study is guided by the Joanna Briggs Institute methodological framework. The search will be conducted on Ovid MEDLINE, CINAHL Plus with Full Text, PsycINFO, and EMBASE, restricting to articles in English. Core concepts of stroke and continuity of care and their synonyms will be used as search terms. Two reviewers will assess articles for inclusion. Data will be extracted and synthesised using quantitative descriptive analysis and deductive content analysis. A steering committee, consisting of people with lived experiences, researchers, and healthcare professionals, will discuss the charted data, and identify any gaps in the literature.<br>Discussion: This research will identify the components of continuity of care interventions, the specific transitions that they address, and any variability in components across different transitions. This research will identify gaps in the continuity of care literature such as where transition interventions are needed.
**Order of Authors:**	Meera Premnazeer
	Sarah Munce
	Mark Bayly
	Kristina Kokorelias
	Monique Gill
	Orianna Scali
	Jill I. Cameron
**Additional Information:**	
**Question**	**Response**
**Financial Disclosure**<br><br>Enter a financial disclosure statement that describes the sources of funding for the work included in this submission. Review the submission guidelines for detailed	Yes

Powered by Editorial Manager® and ProduXion Manager® from Aries Systems Corporation

requirements. View published research articles from *PLOS ONE* for specific examples.<br><br>This statement is required for submission and **will appear in the published article** if the submission is accepted. Please make sure it is accurate.<br><br>**Funded studies**<br>Enter a statement with the following details:<br>• Initials of the authors who received each award<br>• Grant numbers awarded to each author<br>• The full name of each funder<br>• URL of each funder website<br>• Did the sponsors or funders play any role in the study design, data collection and analysis, decision to publish, or preparation of the manuscript?<br><br>Did you receive funding for this work?	
Please add funding details.<br>as follow-up to "**Financial Disclosure**<br><br>Enter a financial disclosure statement that describes the sources of funding for the work included in this submission. Review the submission guidelines for detailed requirements. View published research articles from *PLOS ONE* for specific examples.<br><br>This statement is required for submission and **will appear in the published article** if the submission is accepted. Please make sure it is accurate.	M. Premnazeer is supported by a trainee award from the Rehabilitation Science Research Network for COVID within the Temerity Faculty of Medicine, University of Toronto. M. Premnazeer is also supported through the Ontario Graduate Scholarship, Toronto Rehabilitation Institute Scholarship, and Gwen Bell Endowment Fund from the Rehabilitation Sciences Institute. The funders did not have any role in the study design, data collection and analysis, decision to publish, or preparation of the manuscript.

Powered by Editorial Manager® and ProduXion Manager® from Aries Systems Corporation

**Funded studies**<br>Enter a statement with the following details:<br>• Initials of the authors who received each award<br>• Grant numbers awarded to each author<br>• The full name of each funder<br>• URL of each funder website<br>• Did the sponsors or funders play any role in the study design, data collection and analysis, decision to publish, or preparation of the manuscript?<br><br>Did you receive funding for this work?"	
Please select the country of your main research funder (please select carefully as in some cases this is used in fee calculation).<br>as follow-up to "**Financial Disclosure**<br><br>Enter a financial disclosure statement that describes the sources of funding for the work included in this submission. Review the submission guidelines for detailed requirements. View published research articles from *PLOS ONE* for specific examples.<br><br>This statement is required for submission and **will appear in the published article** if the submission is accepted. Please make sure it is accurate.<br><br>**Funded studies**<br>Enter a statement with the following details:<br>• Initials of the authors who received each award<br>• Grant numbers awarded to each author<br>• The full name of each funder<br>• URL of each funder website<br>• Did the sponsors or funders play any role in the study design, data collection and analysis, decision to publish, or preparation of the manuscript?<br><br>Did you receive funding for this work?"	CANADA - CA
**Competing Interests**	The authors have declared that no competing interests exist.

Powered by Editorial Manager® and ProduXion Manager® from Aries Systems Corporation

Use the instructions below to enter a competing interest statement for this submission. On behalf of all authors, disclose any competing interests that could be perceived to bias this work—acknowledging all financial support and any other relevant financial or non-financial competing interests.<br><br>This statement is required for submission and **will appear in the published article** if the submission is accepted. Please make sure it is accurate and that any funding sources listed in your Funding Information later in the submission form are also declared in your Financial Disclosure statement.<br><br>View published research articles from *PLOS ONE* for specific examples.<br><br>**NO authors have competing interests**<br><br>Enter: *The authors have declared that no competing interests exist.*<br><br>**Authors with competing interests**<br><br>Enter competing interest details beginning with this statement:<br><br>*I have read the journal's policy and the authors of this manuscript have the following competing interests: [insert competing interests here]*<br><br>* typeset	
**Ethics Statement**<br><br>Enter an ethics statement for this submission. This statement is required if the study involved:<br><br>• Human participants<br>• Human specimens or tissue<br>• Vertebrate animals or cephalopods<br>• Vertebrate embryos or tissues	N/A

• Field research<br><br>Write "N/A" if the submission does not require an ethics statement.<br><br>General guidance is provided below. Consult the submission guidelines for detailed instructions. **Make sure that all information entered here is included in the Methods section of the manuscript.**	

Powered by Editorial Manager® and ProduXion Manager® from Aries Systems Corporation

**Format for specific study types**<br><br>**Human Subject Research (involving human participants and/or tissue)**<br>• Give the name of the institutional review board or ethics committee that approved the study<br>• Include the approval number and/or a statement indicating approval of this research<br>• Indicate the form of consent obtained (written/oral) or the reason that consent was not obtained (e.g. the data were analyzed anonymously)<br><br>**Animal Research (involving vertebrate animals, embryos or tissues)**<br>• Provide the name of the Institutional Animal Care and Use Committee (IACUC) or other relevant ethics board that reviewed the study protocol, and indicate whether they approved this research or granted a formal waiver of ethical approval<br>• Include an approval number if one was obtained<br>• If the study involved *non-human primates*, add *additional details* about animal welfare and steps taken to ameliorate suffering<br>• If anesthesia, euthanasia, or any kind of animal sacrifice is part of the study, include briefly which substances and/or methods were applied<br><br>**Field Research**<br><br>Include the following details if this study involves the collection of plant, animal, or other materials from a natural setting:<br>• Field permit number<br>• Name of the institution or relevant body that granted permission	
**Data Availability**<br><br>Authors are required to make the data underlying their research findings fully available, without restriction.<br><br>If the manuscript reports pilot or preliminary results, the data underlying those results must be made available at the time of publication. A Data Availability	N/A - No results are reported

Powered by Editorial Manager® and ProduXion Manager® from Aries Systems Corporation

Statement describing where the data can be found is required at submission. Your answers to this question constitute the Data Availability Statement and will be published in the article, if accepted. When legal or ethical requirements prohibit public data sharing, authors must indicate how researchers may obtain access to the data. See the PLOS Data Policy and FAQ for detailed information.<br><br>**Important:** Stating 'data available on request from the author' is not sufficient. If your data are only available upon request, select 'No' for the first question and explain your situation in the text box.<br><br>For protocols without pilot or preliminary data, authors are strongly encouraged to state how they plan to share research data from their study when it is completed or published.<br><br>If your study includes pilot data, will they be made fully available without restriction?	
**Describe where the data may be found in full sentences. If you are copying our sample text, replace any instances of XXX with the appropriate details.**<br><br>• If **no pilot data are reported** in the manuscript, please enter the following: *No datasets were generated or analysed during the current study. All relevant data from this study will be made available upon study completion.*<br>• If the manuscript reports pilot data **held in a public repository**, include URLs, accession numbers or DOIs. If this information will only be available after acceptance, indicate this by ticking the box below. For example: *All XXX files are available from the XXX database (accession number(s) XXX, XXX.).*<br>• If the manuscript reports pilot data contained **within the manuscript and/or**	No datasets were generated or analysed during the current study. All relevant data from this study will be made available upon study completion.

**Supporting Information files**, enter the following:<br>*All relevant data are within the manuscript and its Supporting Information files.*<br>• If neither of these applies but you are able to provide **details of access elsewhere**, with or without limitations, please do so. For example:<br><br>*Data cannot be shared publicly because of [XXX]. Data are available from the XXX Institutional Data Access / Ethics Committee (contact via XXX) for researchers who meet the criteria for access to confidential data.*<br><br>*The data underlying the results presented in the study are available from (include the name of the third party and contact information or URL).*<br>• This text is appropriate if the data are owned by a third party and authors do not have permission to share the data.<br><br>For study protocols with data management or sharing plans, authors are encouraged to briefly describe in their Data Availability Statement how data they generate will be made accessible when the study is completed. For example:<br>• Deidentified research data will be made publicly available when the study is completed and published.<br><br>* typeset	
Additional data availability information:	

Powered by Editorial Manager® and ProduXion Manager® from Aries Systems Corporation

Manuscript

Click here to access/download;Manuscript;Scoping Review protocol PLOS ONE.docx

Continuity of Care Interventions for People with Stroke and their Caregivers: A Scoping Review Protocol

Meera Premnazeer[1], Sarah Munce[1,2,3], Mark Bayley[1,2,3,4], Kristina Kokorelias[1,5], Monique Gill[1], Orianna Scali[1], Jill I. Cameron[1,2,6*]

1. Rehabilitation Science Institute, Temerty Faculty of Medicine, University of Toronto, Toronto, Ontario, Canada.

2. KITE, Toronto Rehabilitation Institute, Toronto, Ontario, Canada.

3. Institute of Health Policy, Management & Evaluation, University of Toronto, Toronto, Ontario, Canada.

4. Physical Medicine and Rehabilitation, University of Toronto, Toronto, Ontario, Canada.

5. St. John's Rehab Research Program, Sunnybrook Research Institute, Sunnybrook Health Sciences Centre

6. Department of Occupational Science & Occupational Therapy, Temerty Faculty of Medicine, University of Toronto, Toronto, Ontario, Canada.

*Corresponding author

Email: jill.cameron@utoronto.ca

Abstract

Background: The needs of people with stroke and caregivers' change as they transition across the stroke care continuum from initial symptom onset to community living. Continuity of care interventions may support these changing needs. Continuity of care refers to care provided to individuals that is coherent, connected, and consistent across settings. Interventions have been developed to enhance continuity of care, but to date, no reviews have identified the core components of these interventions specific to various care transitions. **Objective:** To examine the literature on continuity of care interventions that address the needs of PWS and/or their caregivers specific to stroke care transitions. **Methods:** The study is guided by the Joanna Briggs Institute methodological framework. The search will be conducted on Ovid MEDLINE, CINAHL Plus with Full Text, PsycINFO, and EMBASE, restricting to articles in English. Core concepts of stroke and continuity of care and their synonyms will be used as search terms. Two reviewers will assess articles for inclusion. Data will be extracted and synthesised using quantitative descriptive analysis and deductive content analysis. A steering committee, consisting of people with lived experiences, researchers, and healthcare professionals, will discuss the charted data, and identify any gaps in the literature. **Discussion:** This research will identify the components of continuity of care interventions, the specific transitions that they address, and any variability in components across different transitions. This research will identify gaps in the continuity of care literature such as where transition interventions are needed.

Introduction

Stroke is a leading cause of disability in Canada (1). It can be associated with a myriad of complications, including difficulty with walking, communicating, and a change in cognitive abilities (2, 3). Due to this complexity, people with stroke (PWS) transition between many elements of the health care system, including acute care hospitals, rehabilitation hospitals, and community care services. As many as 50% of PWS returning to the community have difficulties performing everyday activities, including bathing, housework, and travel (4). PWS require support for everyday activities and transitions between care environments.

In addition to the support PWS receive from the formal health care system, they also receive support from family members and friends (i.e., caregivers) who provide unpaid assistance (5). Caregivers may assist PWS with a variety of daily activities such as cooking or toileting (6). PWS and caregivers receive limited preparation and supports for transitions across settings (e.g., acute care to home) from the formal health care system due to reduced hospital lengths-of-stay (7) and limited continuity across services (8). As a result, caregivers must support individuals with higher levels of disability across fragmented systems of care, while receiving limited support (8-10).

The Canadian Stroke Best Practice Recommendations emphasize the importance of supporting PWS and caregivers as they transition across settings (11). The Canadian Stroke Best Practice Guidelines Transitions of Care Following Stroke Model outlines these transitions (e.g., acute care to rehabilitation) where continuity of care is needed (12). Continuity of care is coherent, connected, and consistent care provided based on a person's needs and personal context as they move from one environment to another (13). Continuity of care has three main dimensions: (1) informational continuity – use of patients' past medical history to make

decisions for present care from one provider to another and/or from one location to another, (2) management continuity – providing timely and consistent care to manage an individual's health needs (i.e., shared management plans across providers), and (3) relational continuity – patient and providers' relationships are continuous (e.g., the same provider provides care over time and across environments) (13, 14). All three dimensions can contribute to better quality of care for PWS and caregivers as they can improve level of communication and trust the patient and caregiver have towards the provider and the healthcare system (13, 15). Exploring continuity of care interventions will help determine the aspects of continuity that have been addressed at different transitions across the stroke care continuum. This review will operationalize continuity of care as interventions that comprise at least one of the three main dimensions outlined above.

The Canadian Stroke Best Practice Recommendations also outline the importance of providing family-centered care as PWS and caregivers transition from one care environment to another (3). A recent scoping review identified key aspects of family-centered care – consideration of patient, family, and care provider collaboration, family contexts, dedicated policies, and illness-specific education in the development and implementation of patient care plans (6). These key aspects were consistent across patient populations, age groups, and care contexts leading to the development of the Universal Model of Family-Centered Care. This model is central to supporting both patients and their caregivers.

Many reviews on continuity of care interventions and stroke have been published (16-19). A recent meta-analysis found that continuity of care interventions supporting the transition from hospital and beyond improved stroke patients' independence compared to usual care (17). A systematic review found low-to-moderate strength of evidence for the effectiveness of hospital-initiated transitional care for PWS or myocardial infarction (18). Another systematic review

found that support interventions to home after acute stroke improved functional status and quality of life in the short-term, and depression and anxiety of PWS in the medium to long-term (16). A literature review on continuity of care interventions for PWS and their caregivers focused on the effectiveness of continuity of care interventions for hospital discharge to the home found some interventions reported positive outcomes (e.g., quality of life, depression and mood) in using continuity of care interventions in patients with stroke (19). The literature review also identified a need to standardize intervention components and their outcomes to identify the most effective interventions. A more recent review examined the inclusion of person-centrered care domains in continuity of care interventions and emphasized the importance of person-centered care in the care of people with stroke (20).

Previous reviews did not aim to comprehensively review all transitions across the care continuum (e.g., stroke onset to the emergency department, inpatient acute care to inpatient rehabilitation). For example, O'Callaghan et al. (2022) compiled continuity of care interventions that supported transitions between hospital to home or inpatient settings to home. The review identified components of continuity of care interventions associated with the single transition from hospital to home or inpatient settings to home (e.g., facilitators of the interventions, method of contract, key strategies used). PWS and caregivers enter the healthcare system much earlier and require continuity of care support from stroke symptom onset as identified by the Canadian Best Practice Recommendations (3).

Reviews to date (16-20) also focused on person-centered care and rarely considered family-centered care (e.g., providing caregivers with illness-specific education) (18)). Incorporating family-centered care components may improve patient, family, caregiver, and health system outcomes (6). An understanding of transition-specific and stroke-specific needs

over time is needed to inform the development of future continuity of care interventions, as PWS and caregivers have differing needs and supports as they move across the care continuum (21). Previous reviews have not focused on the entire stroke care continuum as seen in the Canadian Stroke Best Practice Guidelines Transitions of Care Following Stroke Model and have also not delved into the components identified by the Universal Model of Family-Centered Care.

To date, no reviews have characterized continuity of care interventions for PWS and their caregivers across the entire stroke care continuum and no reviews have explored the provision of family-centered care, specifically. The main objective of this scoping review is to examine the literature on continuity of care interventions that address the needs of PWS and/or their caregivers as they transition. Specific objectives of the scoping review are to:

1. Map the transitions points in the continuum of care that continuity of care interventions address (i.e., transitions found within the Canadian Stroke Best Practice Guidelines Transitions of Care Following Stroke Model (12))
2. Summarize continuity of care models based on the components identified within the Template for Intervention Description and Replication (TIDieR) checklist (22).
3. Summarize and characterize the extent to which domains of continuity of care and family-centered care are included in continuity of care interventions.

A preliminary search for existing reviews on the topic of continuity of care and stroke was conducted on Joanna Briggs Institute (JBI) Database of Systematic Reviews and Implementation Reports, the Cochrane Database of Systematic Reviews, Open Science Framework and MEDLINE in December 2022, February 2023, October 2023, and June 2024. No reviews were identified that met the primary aim of this study. The review has been

registered within Open Science Framework (10.17605/OSF.IO/M2NES). Amendments to the review will be reported within the completed scoping review.

Methods

Design

The scoping review will be conducted following the JBI methodology (23, 24). Reporting will follow the Preferred Reporting Items for Systematic Reviews and Meta-Analyses extension for Scoping Reviews (PRISMA-ScR) (25) and Preferred Reporting Items for Systematic Review and Meta-Analysis Protocols (PRISMA-P) (26).

Steering Committee

A steering committee includes members of the investigative team including two people with lived experience (PWS and caregiver). The investigative team includes individuals who have expertise in stroke, caregiver, and care continuum research (12, 27-29). The team also consists of individuals who have experience in conducting scoping reviews pertaining to PFCC (6, 30-32).

The study objectives and study protocol have been reviewed by experts in the field of stroke (e.g., healthcare professionals who work with PWS and researchers). The steering committee will review, refine, and finalize the research objectives, methodological approaches, results, and dissemination strategies.

Eligibility criteria

Participants

The population of interest includes people with all types of strokes and/or their caregivers (any age). Included studies must have either PWS or their caregivers as participants. Caregivers will be defined as family members or friends who assist with at least one basic (e.g., dressing, toileting) or instrumental (e.g., banking, grocery shopping) activity of daily living per week (33).

Concept

The main concepts of the article will be continuity of care interventions (e.g., the Timing it Right Stroke Family Support Program (34)) for the stroke population. The article should examine at least one transition identified in the Canadian Stroke Best Practice Guidelines Transitions of Care Following Stroke Model (e.g., moving from initial onset to acute care or from acute care to community, long-term care, and palliative care) (11, 12, 27, 35). However, studies that focus on continuity of care interventions for healthcare professionals training to address patient and caregiver needs will be excluded.

Context

Articles describing studies conducted anywhere in the world will be included. The included articles must be written in English. The search criteria will include articles published between January 2000 to present. The restriction to published literature on continuity of care interventions from the year 2000 onwards is to maintain relevance to current practices and ensure consistency with previous reviews (17-19).

Types of Evidence Sources

The review will only include randomized control trials. This was chosen to: 1) ensure feasibility of the review and 2) include high-quality evidence. Specifically, the review will exclude non-randomized controlled trials, quasi-experimental studies, case reports, descriptive cross-sectional studies, and any other experimental and observational quantitative study designs.

The review will also exclude qualitative study designs such as qualitative descriptive, grounded theory, and ethnographic designs. Review articles such as scoping reviews, systematic reviews, and meta-analysis will be used to identify other potential original articles to include in the review that meet the inclusion criteria. Opinion pieces, grey literature, unpublished studies, viewpoints, commentaries, dissertations, theses, book chapters, books, news articles, letters, blogs, editorials, reports, protocol papers and abstracts will not be included.

Search strategy

The research team has developed the search strategy and have consulted a librarian affiliated with the Rehabilitation Sciences Institute at the University of Toronto (See attached search strategy in S1 Appendix A for Ovid MEDLINE Search). Consensus meetings have been held to refine the search strategy. The search strategy will be adapted for each database. The search terms utilized will be consistent with previous reviews conducted on this topic (6, 19). Preliminary searches of Ovid MEDLINE and PsycINFO were conducted to identify additional key search terms commonly found within titles, abstracts, and index terms of relevant studies.

Four electronic databases will be used: (1) Ovid MEDLINE, (2) CINAHL Plus with Full Text, (3) PsycINFO, (4) and EMBASE. These databases were selected as they have previously been used to conduct scoping reviews on continuity of care interventions (6, 13, 19, 36). The research team will examine the reference lists of included articles and previous literature reviews to identify and include articles that meet the inclusion criteria. This method has been known to assist with identifying additional references (13).

Study/Source of Evidence selection

Following the database search, all identified citations will be imported into EndNote and then Covidence where duplicates will be removed (37). Covidence is a platform that supports

screening, reviewing articles, and charting data and has been used previously for scoping reviews (37).

First, a pilot test of the screening process will be conducted with a small subset of articles. Two independent reviewers will screen the title and abstract of 20 articles against the inclusion and exclusion criteria outlined above. The inclusion and exclusion criteria will be modified to enhance clarity between the two reviewers as needed. The pilot test will be repeated in batches of 20 until a Cohen's kappa of 0.80 is achieved. This cut-off has been used in other reviews (38-40). Once a Cohen's kappa of 0.80 is achieved between the two reviewers, the title and abstract screening of the remaining articles will commence.

The same pilot test will be conducted for full-text screening with 20 articles, and it will be repeated if necessary. This will be completed by the same two reviewers and inclusion criteria will be clarified. Full-text screening of the remaining articles will be conducted once a Cohen's kappa of 0.80 is achieved. Reason for exclusion during full-text screening will be recorded and reported in the PRISMA flow diagram of the scoping review. Discrepancies between reviewers during title and abstract or full-text screening will be addressed by a third reviewer or through consensus meetings, if necessary.

As this is a scoping review and not a systematic review, a risk of bias of included studies will not be conducted as it is not a mandatory step and is not often done (41).

Data Extraction

Data will be extracted by two or more independent reviewers using a data extraction tool on Covidence. A pilot test of the full data extraction tool will be completed with a random selection of at least 10 articles. The research team will discuss discrepancies in data extracted

and will modify the data extraction template to enhance consistencies across reviewers. Authors of papers that are included may be contacted to request missing or additional data.

The following descriptive data will be extracted from articles and presented in a summary table (42):

- Author, year of publication, Name of Journal
- Care contexts (setting)
- Country
- Objective and Question(s) of the study
- Study design
- Study population
- Sample size
- Sex and/or gender
- Specific transition (e.g., acute care to home)
- TIDieR checklist describing the interventions (22)
- Components of the intervention as they relate to family-centered care and continuity of care frameworks

The draft extraction tool may be updated as the review progresses. The modifications will be outlined in the scoping review publication.

Data Analysis and Presentation

The scoping review will follow the JBI guidance (2022) to engage in data analysis and presentation through: (1) providing a descriptive numerical summary of the data and deductive content analysis (2) report results by relating to objectives and research questions, and (3) relate the overall findings to implications they have for stroke care (43).

Descriptive numerical summary will be completed using Microsoft Excel (23). This data will summarize the characteristics of included studies using frequencies and percentages (e.g., overall number of studies included, percentage of interventions that address specific transitions) (44).

A deductive content analysis will be used to synthesize qualitative data (43, 45, 46). The review authors will familiarize themselves with the data and extract data according to the main objectives: (1) Mapping continuity of care interventions based on specific transitions across settings (based on Canadian Stroke Best Practice Guidelines Transitions of Care Following Stroke Model (12)); (2) Summarizing components of continuity of care models developed for individual transitions (TIDieR); and (3) Characterizing the extent to which domains of continuity of care and family-centered care are addressed by the interventions. Extracted data will be reviewed by members of the review team to ensure that the information obtained reflects the understanding of the frameworks. The data will be presented as a narrative summary that gives an overall explanation of the findings in relation to the objectives (47).

Co-creation Exercise

Pollock et al. (2022) outline the co-creation exercise as an essential step in the scoping review process for health-related issues. Involving relevant knowledge users in the final review can enhance the findings of the overall review (42, 48). This step will also help to integrate knowledge that may not be accessible within the literature (e.g., new trends that have not been published or historical contexts), increase transparency and rigour, and improve dissemination of findings (48, 49). A virtual meeting will be organized with the steering committee (i.e., investigators, clinicians, program managers, knowledge users in stroke program development and administration, and people with lived experience). This meeting will discuss emerging

themes and identify needs for future practice and research pertaining to stroke care (see S3 Appendix B for interview guide informed from a study on developing questions for focus groups (50)). This co-creation exercise will also be a unique addition as previous scoping reviews of this and related topics did not include this step. This step will also aid in expanding the outreach and dissemination of findings to various knowledge users. Contributions within the co-creation process may provide additional references to be reviewed and identify any gaps in the scoping review (42). Feedback provided during the meeting will be summarized, incorporated into the qualitative thematic analysis and integrated in the overall study outcome (51). Knowledge users who participated in the virtual meeting will be invited to share the findings from the scoping review to the wider community at the end of the study (48).

Ethical considerations

This scoping review is not funded. Institutional Research Ethics Boards at the University of Toronto will not need to review or approve our co-creation exercise as the members will be part of our existing steering committee. There are no conflicts of interest in this research project.

Conclusion

Scoping reviews are identified as one method to inform best practice models (52). This scoping review will be an effective tool to map continuity of care interventions across the stroke care continuum (e.g., acute care to rehab) and is further strengthened by the co-creation exercise.

To date, most reviews on this topic focus on the transition from hospital to home. The purpose of this scoping review is to examine all transitions across the stroke care continuum. This is important as this research will identify gaps in the continuity of care literature such as

where in the care continuum continuity of care interventions have not been studied. This will provide recommendations for future research.

This research will identify and summarize components of continuity of care models that are specific to individual stroke transitions across the entire stroke care continuum as outlined by the Canadian Best Practices Stroke Guidelines Transitions of Care Following Stroke Model (12). As studies from across the world will be included, transitions points not identified by the model will be outlined to inform future models of the stroke care continuum. The review will also summarize and characterize the extent to which domains of continuity of care and family-centered care are addressed in the continuity of care models. Changes that will be made to the scoping review protocol will be reported in the final publication.

The findings from this review will be shared with relevant knowledge users within the steering committee which include developers of the Canadian Best Practices Stroke Guidelines. Findings will also be shared through conferences presentations. The results of the scoping review will be submitted for publication in a scientific journal.

Acknowledgements

M. Premnazeer is supported by a trainee award from the Rehabilitation Science Research Network for COVID within the Temerity Faculty of Medicine, University of Toronto. M. Premnazeer is also supported through the Ontario Graduate Scholarship, Toronto Rehabilitation Institute Scholarship, and Gwen Bell Endowment Fund from the Rehabilitation Sciences Institute. The funders did not have any role in the development of the protocol.

We would like to thank, Katie Merriman, a librarian at the University of Toronto, for their support and consultation in the search strategy for the study.

References

1. Teasell RW, Foley NC, Salter KL, Jutai JW. A blueprint for transforming stroke rehabilitation care in Canada: the case for change. Arch Phys Med Rehabil. 2008;89(3):575-8.
2. Rodgers H, Price C. Stroke unit care, inpatient rehabilitation and early supported discharge. Clin Med (Lond). 2017;17(2):173-7.
3. Cameron JI, O'Connell C, Foley N, Salter K, Booth R, Boyle R, et al. Canadian stroke best practice recommendations: managing transitions of care following stroke, guidelines update 2016. International Journal of Stroke. 2016;11(7):807-22.
4. Mayo NE, Wood-Dauphinee S, Cote R, Durcan L, Carlton J. Activity, participation, and quality of life 6 months poststroke. Arch Phys Med Rehabil. 2002;83(8):1035-42.
5. Bakas T, Clark PC, Kelly-Hayes M, King RB, Lutz BJ, Miller EL. Evidence for stroke family caregiver and dyad interventions: a statement for healthcare professionals from the American Heart Association and American Stroke Association. Stroke. 2014;45(9):2836-52.
6. Kokorelias KM, Gignac MAM, Naglie G, Cameron JI. Towards a universal model of family centered care: a scoping review. BMC Health Serv Res. 2019;19(1):564.
7. Clarke A, Rosen R. Length of stay. How short should hospital care be? Eur J Public Health. 2001;11(2):166-70.
8. Bergman H, Beland F, Lebel P, Contandriopoulos AP, Tousignant P, Brunelle Y, et al. Care for Canada's frail elderly population: fragmentation or integration? CMAJ. 1997;157(8):1116-21.
9. Janzen S, Mirkowski M, McIntyre A, Mehta S, Iruthayarajah J, Teasell R. Referral patterns of stroke rehabilitation inpatients to a model system of outpatient services in Ontario, Canada: a 7-year retrospective analysis. BMC Health Services Research. 2019;19(1):399.
10. Teasell R, Meyer MJ, Foley N, Salter K, Willems D. Stroke Rehabilitation in Canada: A Work in Progress. Topics in Stroke Rehabilitation. 2009;16(1):11-9.
11. Mountain A, Patrice Lindsay M, Teasell R, Salbach NM, de Jong A, Foley N, et al. Canadian Stroke Best Practice Recommendations: Rehabilitation, Recovery, and Community Participation following Stroke. Part Two: Transitions and Community Participation Following Stroke. Int J Stroke. 2020:1747493019897847.
12. Cameron JI, O'Connell C, Foley N, Salter K, Booth R, Boyle R, et al. Canadian Stroke Best Practice Recommendations: Managing transitions of care following Stroke, Guidelines Update 2016. Int J Stroke. 2016;11(7):807-22.
13. Haggerty JL, Reid RJ, Freeman GK, Starfield BH, Adair CE, McKendry R. Continuity of care: a multidisciplinary review. Bmj. 2003;327(7425):1219-21.
14. Uijen AA, Schers HJ, Schellevis FG, van den Bosch WJ. How unique is continuity of care? A review of continuity and related concepts. Fam Pract. 2012;29(3):264-71.
15. Facchinetti G, D'Angelo D, Piredda M, Petitti T, Matarese M, Oliveti A, et al. Continuity of care interventions for preventing hospital readmission of older people with chronic diseases: A meta-analysis. Int J Nurs Stud. 2020;101:103396.
16. O'Callaghan G, Fahy M, Murphy P, Langhorne P, Galvin R, Horgan F. Effectiveness of interventions to support the transition home after acute stroke: a systematic review and meta-analysis. BMC Health Services Research. 2022;22(1).

17. Wang Y, Yang F, Shi H, Yang C, Hu H. What Type of Transitional Care Effectively Reduced Mortality and Improved ADL of Stroke Patients? A Meta-Analysis. Int J Environ Res Public Health. 2017;14(5).
18. Prvu Bettger J, Alexander KP, Dolor RJ, Olson DM, Kendrick AS, Wing L, et al. Transitional care after hospitalization for acute stroke or myocardial infarction: a systematic review. Ann Intern Med. 2012;157(6):407-16.
19. Puhr MI, Thompson HJ. The Use of Transitional Care Models in Patients With Stroke. J Neurosci Nurs. 2015;47(4):223-34.
20. Nelson MLA, MacEachern E, Prvu Bettger J, Camicia M, García JJ, Kapral MK, et al. Exploring the Inclusion of Person-Centered Care Domains in Stroke Transitions of Care Interventions: A Scientific Statement From the American Heart Association. Stroke. 2024;55(6):e169-e81.
21. Cameron JI, Naglie G, Silver FL, Gignac MA. Stroke family caregivers' support needs change across the care continuum: a qualitative study using the timing it right framework. Disabil Rehabil. 2013;35(4):315-24.
22. Hoffmann TC, Glasziou PP, Boutron I, Milne R, Perera R, Moher D, et al. Better reporting of interventions: template for intervention description and replication (TIDieR) checklist and guide. BMJ : British Medical Journal. 2014;348:g1687.
23. Peters M, Godfrey C, McInerney P, Munn Z, Tricco A, Khalil H. 2022 2022. In: JBI Manual for Evidence Synthesis [Internet]. Available from: https://synthesismanual.jbi.global.
24. Peters MDJ, Godfrey C, McInerney P, Khalil H, Larsen P, Marnie C, et al. Best practice guidance and reporting items for the development of scoping review protocols. JBI Evid Synth. 2022;20(4):953-68.
25. Tricco AC, Lillie E, Zarin W, O'Brien KK, Colquhoun H, Levac D, et al. PRISMA extension for scoping reviews (PRISMA-ScR): checklist and explanation. Ann Intern Med. 2018;169(7):467-73.
26. Shamseer L, Moher D, Clarke M, Ghersi D, Alessandro Liberati, Petticrew M, et al. Preferred reporting items for systematic review and meta-analysis protocols (PRISMA-P) 2015: elaboration and explanation. BMJ : British Medical Journal. 2015;349:g7647.
27. Cameron JI, Gignac MA. "Timing It Right": A Conceptual Framework for Addressing the Support Needs of Family Caregivers to Stroke Survivors from the Hospital to the Home. Patient Education & Counseling. 2008;70:305-14.
28. Cameron JI, Naglie G, Green TL, Gignac MA, Bayley M, Huijbregts M, et al. A feasibility and pilot randomized controlled trial of the "Timing it Right Stroke Family Support Program". Clin Rehabil. 2015;29(11):1129-40.
29. Cameron JI, Naglie G, Gignac MA, Bayley M, Warner G, Green T, et al. Randomized clinical trial of the Timing it Right Stroke Family Support Program: research protocol. BMC Health Serv Res. 2014;14:18.
30. Kokorelias KM, Cameron JI, Salbach NM, Colquhoun H, Munce SEP, Nelson MLA, et al. Exploring the Poststroke Experiences and Needs of South Asian Communities Living in High-Income Countries: Findings from a Scoping Review. Journal of Racial and Ethnic Health Disparities. 2023.
31. Munce SEP, Shepherd J, Perrier L, Allin S, Sweet SN, Tomasone JR, et al. Online peer support interventions for chronic conditions: a scoping review protocol. BMJ Open. 2017;7(9):e017999.

32. McDougall A, Bayley M, Munce SEP. The ketogenic diet as a treatment for traumatic brain injury: a scoping review. Brain Injury. 2018;32(4):416-22.
33. Smolej E, Malozewski M, McKendry S, Diab K, Daubert C, Farnum A, et al. A qualitative study exploring family caregivers' support needs in the context of medical assistance in dying. Palliative and Supportive Care. 2022;21:1-7.
34. Cameron JI, Naglie G, Green TL, Gignac MA, Bayley M, Huijbregts M, et al. A feasibility and pilot randomized controlled trial of the "Timing it Right Stroke Family Support Program". Clinical Rehabilitation. 2015;29(11):1129-40.
35. Lanctôt KL, Lindsay MP, Smith EE, Sahlas DJ, Foley N, Gubitz G, et al. Canadian Stroke Best Practice Recommendations: Mood, Cognition and Fatigue following Stroke, 6th edition update 2019. International Journal of Stroke. 2019;15(6):668-88.
36. Liebzeit D, Rutkowski R, Arbaje AI, Fields B, Werner NE. A scoping review of interventions for older adults transitioning from hospital to home. J Am Geriatr Soc. 2021;69(10):2950-62.
37. Covidence - Better systematic review management. Covidence.
38. Pouwels S, Stokmans RA, Willigendael EM, Nienhuijs SW, Rosman C, van Ramshorst B, et al. Preoperative exercise therapy for elective major abdominal surgery: a systematic review. International Journal of Surgery. 2014;12(2):134-40.
39. Damanhoury S, Newton A, Rashid M, Hartling L, Byrne J, Ball G. Defining metabolically healthy obesity in children: a scoping review. Obesity Reviews. 2018;19(11):1476-91.
40. Colquhoun HL, Jesus TS, O'Brien KK, Tricco AC, Chui A, Zarin W, et al. Scoping Review on Rehabilitation Scoping Reviews. Arch Phys Med Rehabil. 2020;101(8):1462-9.
41. Peters MDJ, Marnie C, Colquhoun H, Garritty CM, Hempel S, Horsley T, et al. Scoping reviews: reinforcing and advancing the methodology and application. Systematic Reviews. 2021;10(1):263.
42. Arksey H, O'Malley L. Scoping studies: towards a methodological framework. International Journal of Social Research Methodology. 2005;8(1):19-32.
43. Pollock D, Peters MDJ, Khalil H, McInerney P, Alexander L, Tricco AC, et al. Recommendations for the extraction, analysis, and presentation of results in scoping reviews. JBI Evid Synth. 2023;21(3):520-32.
44. Pham MT, Rajić A, Greig JD, Sargeant JM, Papadopoulos A, McEwen SA. A scoping review of scoping reviews: advancing the approach and enhancing the consistency. Res Synth Methods. 2014;5(4):371-85.
45. Mayring P. Qualitative Content Analysis: Theoretical Background and Procedures. In: Bikner-Ahsbahs A, Knipping C, Presmeg N, editors. Approaches to Qualitative Research in Mathematics Education: Examples of Methodology and Methods. Dordrecht: Springer Netherlands; 2015. p. 365-80.
46. Elo S, Kyngäs H. The qualitative content analysis process. J Adv Nurs. 2008;62(1):107-15.
47. Vears DF, Gillam L. Inductive content analysis: A guide for beginning qualitative researchers. Focus on Health Professional Education: A Multi-Professional Journal. 2022;23(1):111-27.
48. Pollock D, Alexander L, Munn Z, Peters MDJ, Khalil H, Godfrey CM, et al. Moving from consultation to co-creation with knowledge users in scoping reviews: guidance from the JBI Scoping Review Methodology Group. JBI Evidence Synthesis. 2022;20(4).

49. Westphaln KK, Regoeczi W, Masotya M, Vazquez-Westphaln B, Lounsbury K, McDavid L, et al. From Arksey and O'Malley and Beyond: Customizations to enhance a team-based, mixed approach to scoping review methodology. MethodsX. 2021;8:101375.
50. Krueger RA, Casey MA. Designing and conducting focus group interviews: Citeseer; 2002.
51. Levac D, Colquhoun H, O'Brien KK. Scoping studies: advancing the methodology. Implementation Science. 2010;5(1):69.
52. Munn Z, Peters MDJ, Stern C, Tufanaru C, McArthur A, Aromataris E. Systematic review or scoping review? Guidance for authors when choosing between a systematic or scoping review approach. BMC Medical Research Methodology. 2018;18(1):143.

Supporting information

S1 Appendix A. This is the Search Strategy for MEDLINE via Ovid.

S2 Appendix B. This is the Draft Interview Guide for the Co-Creation Exercise.

S3 Appendix C. This is the PRISMA-P 2015 Checklist.

Click here to access/download
Supporting Information
S1_Appendix A.docx

Click here to access/download
Supporting Information
S2_Appendix B.docx

Click here to access/download
Supporting Information
S3_Appendix C.docx
