# Supplementary material for: Continuity of care interventions for people with stroke and their caregivers: A scoping review protocol
Source: PLoS One. 2025 May 9;20(5):e0323344. doi: 10.1371/journal.pone.0323344 (PMC12063880; doi:10.1371/journal.pone.0323344)
Supplement: Attachment — Submitted filename: Comments.docx [file pone.0323344.s005.docx]

The manuscript presents an exceptionally well-organized and thorough study protocol that addresses an important gap in stroke care research, specifically in continuity of care interventions for people with stroke (PWS) and their caregivers. The work demonstrates a deep understanding of both the clinical and research landscapes, and the methodology is meticulously designed to ensure a comprehensive scoping review. The authors have successfully articulated the relevance of their study, which will likely contribute significantly to improving stroke care transitions.

However, there are some areas for improvement that could enhance the clarity and impact of the manuscript. In the **abstract**, while the objectives and methods are clearly stated, further detail could be added to the methods section to outline the databases and search terms more explicitly. This would provide readers with a clearer understanding of the scope of the research at a glance. The **introduction** is comprehensive but could benefit from being more concise, with a more direct focus on the specific research gap. Some redundancy in the background could be streamlined to improve flow and reader engagement. In the **methodology**, the choice to limit the review to randomized controlled trials could be reconsidered, as scoping reviews typically aim to include a broader range of evidence types. Expanding the types of studies included would align more closely with the objectives of mapping the breadth of available evidence on continuity of care interventions.

Overall, the manuscript showcases a high level of scholarly rigor and has great potential for advancing research in stroke care. With these small adjustments, the work can be further strengthened to ensure maximum impact and clarity.
